# Prognostic performance of serum YKL-40 for one-year clinical outcomes in acute ischemic stroke

**DOI:** 10.18632/aging.204553

**Published:** 2023-02-25

**Authors:** Guomei Shi, Minghao Li, Yan E, Meng Wang, Pengyu Gong, Xiaorong Wang, Jingye Lu, Weixiang Wu, Shouru Xue, Junshan Zhou, Rujuan Zhou

**Affiliations:** 1 Department of Neurology, The Taixing People’s Hospital, Taixing 225400, Jiangsu Province, China; 2 Department of Neurology, The First Affiliated Hospital of Soochow University, Suzhou 215006, Jiangsu Province, China; 3 Department of Vascular Surgery, The Taixing People’s Hospital, Taixing 225400, Jiangsu Province, China; 4 Department of Neurology, Nanjing First Hospital, Nanjing Medical University, Nanjing 210001, Jiangsu Province, China

**Keywords:** YKL-40, acute ischemic stroke, outcome, mortality, recurrence

## Abstract

Background: Effects of YKL-40 on one-year clinical outcomes including poor clinical outcome, all-cause mortality, and stroke recurrence among acute ischemic stroke (AIS) patients remained elusive. The purpose of this study was to explore the association between serum YKL-40 at admission and one-year clinical outcomes in AIS patients.

Methods: In this prospective cohort study, a total of 1002 participants out of 1361 AIS patients from two centers were included for current analysis. Serum YKL-40 concentrations were measured via enzyme-linked immunosorbent assay. Multivariable logistic or Cox regression were performed to explore the independent association of YKL-40 with one-year clinical outcomes, including poor outcome (modified Rankin Scale of 3-6), all-cause mortality, and recurrent stroke. C-statistic, net reclassification index (NRI) and integrated discrimination improvement (IDI) were calculated to evaluate the discriminatory and predictive power of YKL-40 when added to conventional model.

Results: Compared with the first quartile of YKL-40, the adjusted odds ratios or hazard ratios with 95% confidence intervals of the fourth quartile were 3.032 (1.627-5.650) for poor outcome, 2.886 (1.320-6.308) for all-cause mortality and 1.694 (0.906-3.169) for recurrent stroke. The addition of serum YKL-40 to conventional model significantly improved reclassification for poor outcome (NRI 0.053, P = 0.031; IDI 0.018, P = 0.001) and all-cause mortality (NRI 0.162, P = 0.036).

Conclusions: Elevated serum YKL-40 at admission might be independently associated with one-year poor outcome and all-cause mortality but not stroke recurrence among Chinese AIS patients.

## INTRODUCTION

Acute ischemic stroke (AIS) is reported to be a serious global public health problem, which is among the leading reasons for mortality and long-term disability [1, 2]. Despite widespread use of effective interventions such as intravenous thrombolysis, endovascular treatment and secondary prevention therapy, the residual risk of long-term disability and mortality remained substantial [1, 2]. Traditional prognostic factors do not explain all risks of poor clinical outcomes, and an early risk assessment with strong ability of estimating the prognosis of AIS is crucial and clinically valuable.

A growing body of evidence suggests that inflammatory processes play an extraordinary role in the pathophysiology of AIS and affect the prognosis of AIS [3, 4]. YKL-40, also called chitinase-3-like-1 protein (CHI3L1) or breast regression protein 39 (BRP-39), is a novel inflammatory factor and has been shown to be involved in angiogenesis, tissue fibrosis, inflammation, oxidative tissue injury, and extracellular remodeling responses [5]. Elevated levels of YKL-40 were reported to be associated with unstable carotid plaque [6], increased risk for ischemic stroke [7], as well as stroke-associated pneumonia (SAP) [8]. To date, several clinical studies with small sample sizes have demonstrated that YKL-40 was related to clinical outcomes of AIS patients. However, controversy still existed regarding the influence of YKL-40 on stroke recurrence [9, 10]. Moreover, it is known that SAP is a standout complication after AIS and leads to poor clinical outcomes [11], nevertheless, there is limited data evaluating the prognostic role of YKL-40 in clinical outcomes with SAP in AIS patients.

In this hospital-based prospective study, we aimed to investigate the role of serum YKL-40 at admission in the prediction of one-year clinical outcomes, including poor outcome, all-cause mortality, and recurrent stroke, in patients with AIS.

## RESULTS

### Baseline characteristics

**Figure 1:**
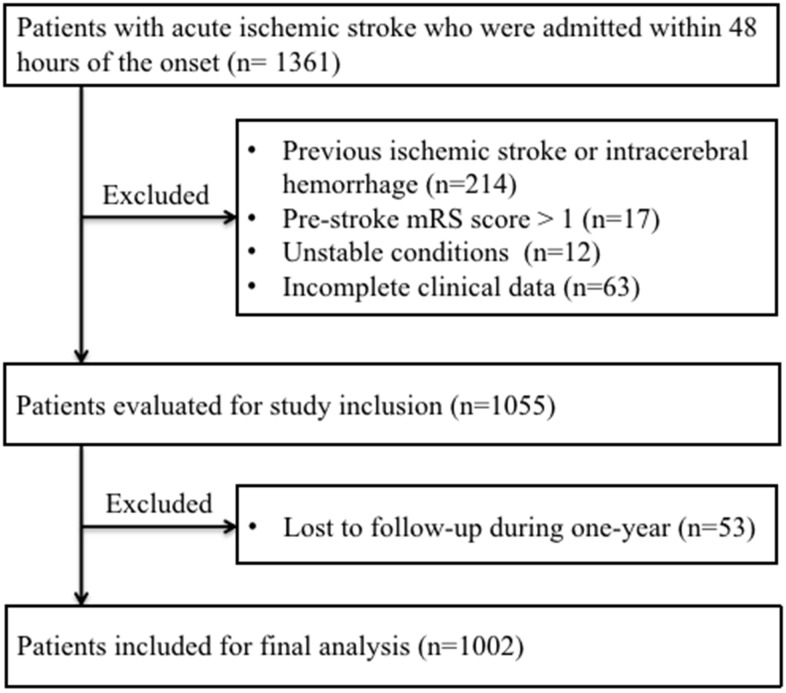
**Patient flowchart.** Abbreviations: mRS, modified Rankin Scale.

Between February 2020 and March 2021, a total of 1361 AIS patients who met the criteria and provided written informed consent were consecutively enrolled in this study. Three hundred and fifty-nine AIS patients were excluded for the reasons below: previous ischemic stroke or intracerebral hemorrhage (n=214); pre-stroke modified Rankin Scale (mRS) score > 1 (n=17); unstable conditions (n=12); incomplete clinical data (n=63); lost to follow-up at one-year (n=53). Ultimately, 1002 patients were included in final analysis, including 619 (61.8%) males and 383 (39.2%) females, and the median age was 70 (interquartile ranges [IQR], 60-78) years (Figure 1).

Patients were divided into four groups according to quartiles of YKL-40: first quartile, 23.6-83.5 ng/mL; second quartile, 83.9-138.6 ng/mL; third quartile, 139.6-245.1 ng/mL; fourth quartile, 245.2-798.0 ng/mL. Baseline characteristics of participants stratified by YKL-40 quartiles were summarized in Table 1. The variables with significant differences among four groups were presented below: age, coronary artery disease, atrial fibrillation, drinking alcohol, baseline National Institutes of Health Stroke Score (NIHSS), dysphagia, SAP, Trial of Org 10172 in Acute Stroke Treatment (TOAST) subtype, leukocyte, triglyceride (TG), high-density lipoprotein (HDL), fasting blood glucose (FBG), as well as high sensitivity C-reactive protein (hs-CRP) (*P* < 0.05 for all). In terms of one-year clinical outcomes, the incidence rates of poor outcome, all-cause mortality, and recurrent stroke were 25.8%, 13.4%, and 10.4%, respectively. The rates of poor outcome (*P* = 0.001) and all-cause mortality (*P* = 0.001) increased by YKL-40 quartiles, whereas recurrent stroke was not statistically significant different among four groups (*P* = 0.070).

**Table 1:** Baseline characteristics of patients stratified by quartiles of YKL-40.

**Variable**	**Total (n=1002)**	**First quartile (n=251)**	**Second quartile (n=251)**	**Third quartile (n=250)**	**Fourth quartile (n=250)**	***P* value**
Demographic characteristics						
Age (years), median (IQR)	70 (60, 78)	68 (58, 74)	68 (58, 78)	70 (61, 79)	75 (63, 83)	0.001
Male, *n* (%)	619 (61.8)	171 (68.1)	151 (60.2)	151 (60.4)	146 (58.4)	0.113
Past medical history, *n* (%)						
Hypertension	699 (69.8)	179 (71.3)	174 (69.3)	172 (68.8)	174 (69.6)	0.936
Diabetes mellitus	216 (21.6)	58 (23.1)	53 (21.1)	46 (18.4)	59 (23.6)	0.480
Dyslipidemia	136 (13.6)	30 (12.0)	31 (12.4)	40 (16.0)	35 (14.0)	0.537
Coronary artery disease	137 (13.7)	27 (10.8)	30 (12.0)	32 (12.8)	48 (19.2)	0.028
Atrial fibrillation	169 (16.9)	31 (12.4)	30 (12.0)	54 (21.6)	54 (21.6)	0.001
Smoking						0.385
Never	575 (57.4)	138 (55.0)	142 (56.6)	143 (57.2)	152 (60.8)	
Ever smoking	310 (30.9)	76 (30.3)	80 (31.9)	85 (34.0)	69 (27.6)	
Currently smoking	117 (11.7)	37 (14.7)	29 (11.6)	22 (8.8)	29 (11.6)	
Drinking alcohol						0.017
Never	645 (64.4)	155 (61.8)	164 (65.3)	156 (62.4)	170 (68.0)	
Ever drinking alcohol	248 (24.8)	57 (22.7)	55 (21.9)	74 (29.6)	62 (24.8)	
Currently drinking alcohol	109 (10.9)	39 (15.5)	32 (12.7)	20 (8.0)	18 (7.2)	
Clinical assessment						
Baseline NIHSS, median (IQR)	4 (2, 11)	3 (2, 5)	4 (2, 6)	6 (3, 13)	10 (4, 14)	0.001
Intravenous thrombolysis, *n* (%)	341 (34.0)	76 (30.3)	84 (33.5)	89 (35.6)	92 (36.8)	0.434
Dysphagia, *n* (%)	300 (29.9)	61 (24.3)	64 (25.5)	75 (30.0)	100 (40.0)	0.001
SAP, *n* (%)	274 (27.3)	21 (8.4)	45 (17.9)	72 (28.8)	136 (54.4)	0.001
Stroke subtype, *n* (%)						0.001
LAA	469 (46.8)	105 (41.8)	123 (49.0)	114 (45.6)	127 (50.8)	
CE	200 (20.0)	39 (15.5)	28 (11.2)	65 (26.0)	68 (27.2)	
SAO	281 (27.8)	99 (39.4)	82 (32.7)	62 (24.8)	38 (15.2)	
SOE	15 (1.5)	3 (1.2)	2 (0.8)	4 (1.6)	6 (2.4)	
SUE	37 (3.7)	5 (2.0)	16 (6.4)	5 (2.0)	11 (4.4)	
Laboratory data, median (IQR)						
Leukocyte (10^^9^/L)	7.62 (6.07, 9.52)	7.42 (5.94, 9.10)	7.19 (5.74, 8.65)	7.99 (6.21, 9.85)	8.32 (6.47, 10.34)	0.001
TC (mmol/L)	4.46 (3.89, 5.26)	4.64 (4.07, 5.26)	4.33 (3.89, 5.26)	4.49 (3.84, 5.28)	4.38 (3.82, 5.25)	0.248
TG (mmol/L)	1.22 (0.87, 1.77)	1.35 (0.94, 1.90)	1.30 (0.95, 1.95)	1.18 (0.88, 1.64)	1.08 (0.77, 1.54)	0.001
HDL (mmol/L)	1.14 (0.95, 1.36)	1.10 (0.91, 1.34)	1.12 (0.91, 1.34)	1.14 (0.99, 1.38)	1.18 (1.00, 1.38)	0.030
LDL (mmol/L)	2.57 (2.03, 3.19)	2.77 (2.04, 3.30)	2.56 (2.03, 3.15)	2.55 (2.13, 3.23)	2.48 (1.94, 3.33)	0.449
FBG (mmol/L)	5.75 (4.98, 7.24)	5.74 (5.02, 7.20)	5.51 (4.84, 6.99)	5.60 (4.81, 6.73)	6.44 (5.18, 8.09)	0.001
Homocysteine (μmol/L)	14.40 (12.00, 17.82)	14.57 (12.00, 16.95)	14.08 (11.90, 17.58)	14.63 (12.91, 19.02)	14.52 (11.86, 17.84)	0.261
Hs-CRP (mg/L)	3.55 (1.28, 7.81)	2.12 (0.88, 5.06)	2.48 (0.80, 6.87)	3.98 (1.59, 8.94)	5.81 (2.67,12.76)	0.001
One-year clinical outcomes, *n* (%)						
Poor outcome	259 (25.8)	25 (10.0)	39 (15.5)	75 (30.0)	120 (48.0)	0.001
All-cause mortality	134 (13.4)	4 (1.6)	14 (5.6)	48 (19.2)	68 (27.2)	0.001
Recurrent stroke	104 (10.4)	20 (8.0)	21 (8.4)	27 (10.8)	36 (14.4)	0.070

Abbreviations: IQR, inter quartile range; NIHSS, National Institute of Health Stroke Scale; SAP, stroke-associated pneumonia; TOAST, Trial of Org 10172 in Acute Stroke Treatment; LAA, large-artery atherosclerosis; CE, cardioembolism; SAO, small-artery occlusion; SOE, stroke of other determined etiology; SUE, stroke of undetermined etiology; TC, total cholesterol; TG, triglyceride; HDL, high-density lipoprotein; LDL, low-density lipoprotein; FBG, fasting blood glucose; Hs-CRP, high sensitivity C-reactive protein.

### YKL-40 and one-year clinical outcomes

**Figure 2:**
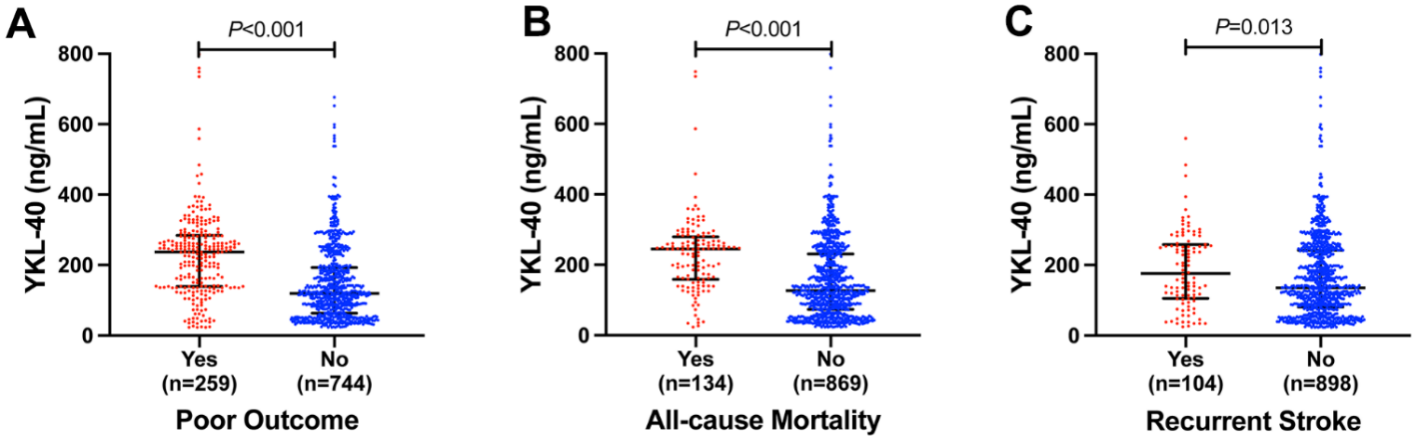
Serum YKL-40 levels in different groups according to (**A**) poor outcome, (**B**) all-cause mortality and (**C**) recurrent stroke in AIS patients. Mann-Whitney U Test. Horizontal lines represent medians and interquartile ranges (IQRs).

**Figure 3:**
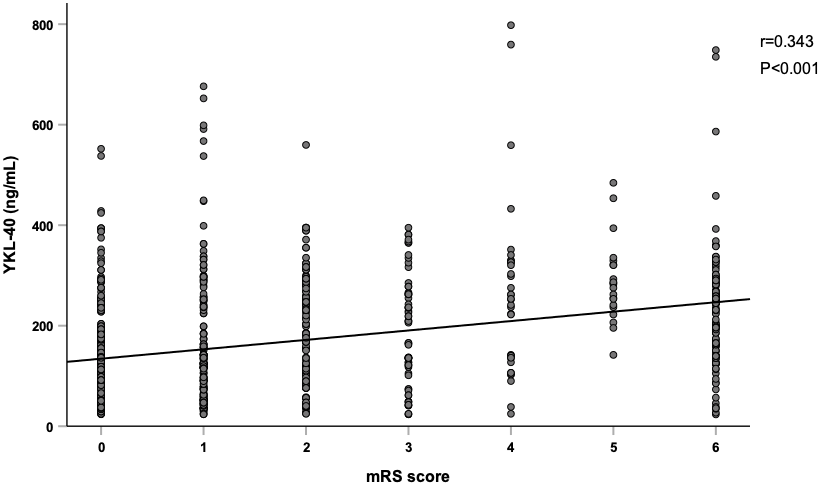
**Correlation between YKL-40 and mRS score.** YKL-40 showed a significant relationship with modified Rankin Scale (mRS) score. (r=0.343, *P*<0.001, and Spearman rank correlation analysis).

Figure 2 showed serum YKL-40 levels in different groups according to one-year clinical outcomes. YKL-40 levels were higher in patients with poor outcome (237.0 [139.5, 284.1] versus 119.6 [63.5, 193.0] ng/mL, *P* < 0.001), all-cause mortality (244.9 [159.1, 279.5] versus 126.7 [73.9, 230.8] ng/mL, *P* < 0.001), and recurrent stroke (176.2 [105.8, 258.1] versus 135.8 [81.3, 242.1] ng/mL, *P* = 0.013). Moreover, YKL-40 levels showed a limited correlation with mRS score at one-year (r = 0.343, *P* < 0.001) (Figure 3).

Table 2 demonstrated the crude and adjusted odds ratios (ORs) or hazard ratios (HRs) with 95% confidence intervals (CIs) of YKL-40 levels for one-year clinical outcomes. In the unadjusted model, when comparing with the first quartile of YKL-40, the third and fourth quartiles were independently associated with poor outcome (OR 3.874, 95% CI 2.365-6.348 for the third quartile; OR 8.345, 95% CI 5.154-13.511 for the fourth quartile) as well as all-cause mortality (HR 6.022, 95% CI 2.838-12.774 for the third quartile; HR 9.354, 95% CI 4.490-19.487 for the fourth quartile), and the fourth quartile was independently associated with recurrent stroke (HR 1.884, 95% CI 1.091-3.255). Furthermore, after adjustment for potential confounders in Model 3, the fourth quartile remained significantly associated with poor outcome (OR 3.032, 95% CI 1.627-5.650), the third and fourth quartiles remained independently associated with all-cause mortality (HR 2.356, 95% CI 1.067-5.201 for the third quartile; HR 2.886, 95% CI 1.320-6.308 for the fourth quartile), while the significant association between the fourth quartile with recurrent stroke disappeared (HR 1.694, 95% CI 0.906-3.169).

**Table 2:** Associations of YKL-40 levels with one-year clinical outcomes.

**YKL-40**	**Poor outcome**		**All-cause mortality**		**Recurrent stroke**	
	**OR (95% CI)**	***P* value**	**HR (95% CI)**	***P* value**	**HR (95% CI)**	***P* value**
Unadjusted						
First quartile	1.000 (reference)		1.000 (reference)		1.000 (reference)	
Second quartile	1.663 (0.973-2.842)	0.063	1.914 (0.811-4.513)	0.138	1.058 (0.574-1.953)	0.856
Third quartile	3.874 (2.365-6.348)	0.001	6.022 (2.838-12.774)	0.001	1.377 (0.772-2.455)	0.279
Fourth quartile	8.345 (5.154-13.511)	0.001	9.354 (4.490-19.487)	0.001	1.884 (1.091-3.255)	0.023
Model 1						
First quartile	1.000 (reference)		1.000 (reference)		1.000 (reference)	
Second quartile	1.498 (0.849-2.643)	0.163	1.679 (0.711-3.964)	0.237	0.964 (0.522-1.781)	0.906
Third quartile	3.546 (2.092-6.010)	0.001	4.837 (2.274-10.289)	0.001	1.181 (0.661-2.112)	0.575
Fourth quartile	6.669 (3.975-11.186)	0.001	6.085 (2.903-12.754)	0.001	1.371 (0.786-2.393)	0.266
Model 2						
First quartile	1.000 (reference)		1.000 (reference)		1.000 (reference)	
Second quartile	1.131 (0.589-2.174)	0.711	1.308 (0.546-3.136)	0.547	1.294 (0.684-2.449)	0.428
Third quartile	1.725 (0.922-3.225)	0.088	2.250 (1.030-4.913)	0.042	1.334 (0.712-2.498)	0.368
Fourth quartile	2.856 (1.559-5.230)	0.001	2.674 (1.238-5.776)	0.012	1.569 (0.855-2.881)	0.146
Model 3						
First quartile	1.000 (reference)		1.000 (reference)		1.000 (reference)	
Second quartile	1.104 (0.564-2.162)	0.772	1.222 (0.501-2.982)	0.659	1.373 (0.717-2.629)	0.339
Third quartile	1.741 (0.922-3.287)	0.087	2.356 (1.067-5.201)	0.034	1.335 (0.708-2.518)	0.372
Fourth quartile	3.032 (1.627-5.650)	0.001	2.886 (1.320-6.308)	0.008	1.694 (0.906-3.169)	0.099

Model 1: Adjusted for age and gender; Model 2: Adjusted for Model 1 + history of hypertension, diabetes mellitus, hyperlipidemia, coronary artery disease, atrial fibrillation, smoking, drinking alcohol, baseline NIHSS, intravenous thrombolysis, dysphagia, SAP, and TOAST subtype. Model 3: Model 2 + leukocyte, TC, TG, HDL, LDL, FBG, homocysteine and hs-CRP.

Abbreviations: OR, odd ratio; HR, hazard ratio; CI, confidence interval; NIHSS, National Institute of Health Stroke Scale; SAP, stroke-associated pneumonia; TOAST, Trial of Org 10172 in Acute Stroke Treatment; TC, total cholesterol; TG, triglyceride; HDL, high-density lipoprotein; LDL, low-density lipoprotein; FBG, fasting blood glucose; hs-CRP, high sensitivity C-reactive protein.

### Subgroup analysis for YKL-40 and one-year clinical outcomes

**Figure 4:**
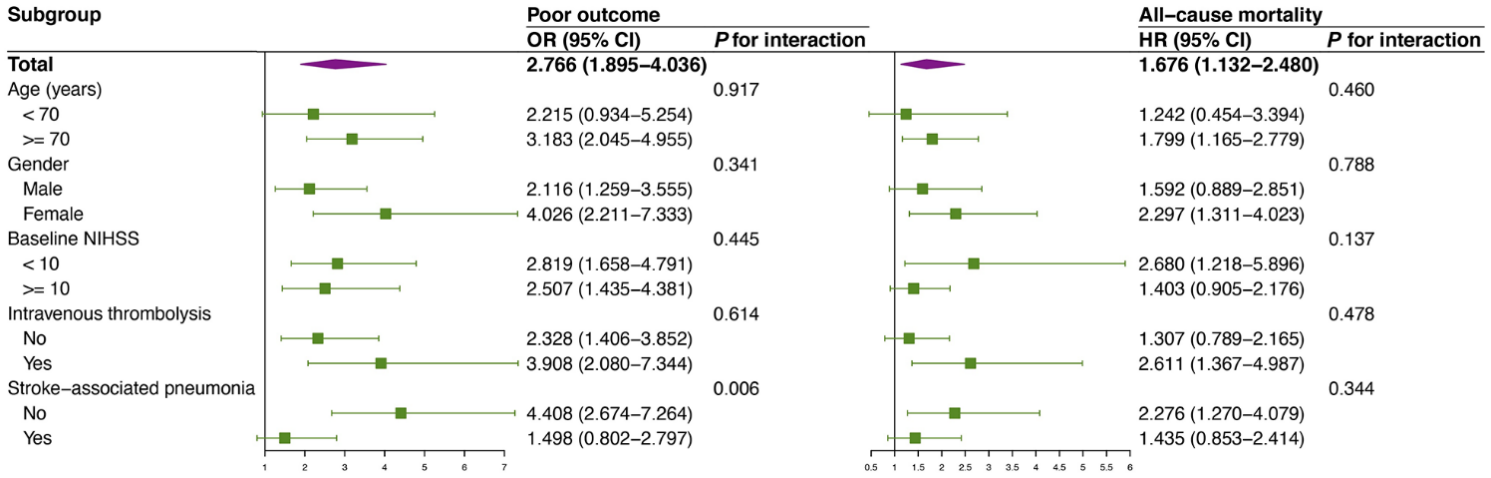
**Subgroup analyses of the association between baseline YKL-40 and poor outcome as well as all-cause mortality.** Interactions between baseline YKL-40 and interesting factors on one-year clinical outcomes were tested by the likelihood ratio test with adjustment for age, gender, history of hypertension, diabetes mellitus, hyperlipidemia, coronary artery disease, atrial fibrillation, smoking, drinking alcohol, baseline NIHSS, intravenous thrombolysis, and SAP other than variable was used as a subgroup. Abbreviations: OR, odd ratio; HR, hazard ratio; CI, confidence interval; NIHSS, National Institute of Health Stroke Scale; SAP, stroke-associated pneumonia.

In addition, subgroup analyses for the associations of serum YKL-40 and one-year clinical outcomes stratified by age (< 70 versus ≥ 70), gender (male versus female), baseline NIHSS (< 10 versus ≥ 10), intravenous thrombolysis (no versus yes), and SAP (no versus yes) were depicted in Figure 4. The effects of YKL-40 on poor outcome and all-cause mortality were consistent with respect to age, gender, baseline NIHSS, and intravenous thrombolysis (*P* for interaction > 0.05 for all). However, a significant interaction was observed between YKL-40 and SAP for poor outcome (*P* for interaction = 0.006). YKL-40 increased the risk of one-year poor outcome in the subgroup without SAP (OR 4.408, 95% CI 2.674-7.264).

### Incremental predictive value of YKL-40

We further assessed whether YKL-40 would increase the predictive value of conventional risk factors including age, gender, history of hypertension, diabetes mellitus, hyperlipidemia, coronary artery disease, atrial fibrillation, smoking, drinking alcohol, baseline NIHSS, intravenous thrombolysis, SAP, and TOAST subtype (Table 3). The addition of YKL-40 to conventional model did not remarkably improve the C-statistics for poor outcome (*P* = 0.653), all-cause mortality (*P* = 0.985), or recurrent stroke (*P* = 0.175). However, adding YKL-40 to conventional model did significantly improve risk reclassification for poor outcome (net reclassification index [NRI] 0.053, 95% CI 0.005-0.101, *P* = 0.031; integrated discrimination improvement [IDI] 0.018, 95% CI 0.009-0.027, *P* = 0.001) and all-cause mortality (NRI 0.162, 95% CI 0.024-0.265, *P* = 0.036).

**Table 3:** Incremental predictive value of YKL-40 for one-year clinical outcomes.

**YKL-40**	**Discrimination**		**Reclassification**			
	**C-statistic (95% CI)**	***P* value**	**NRI (95% CI)**	***P* value**	**IDI (95% CI)**	***P* value**
Poor outcome						
Conventional model	0.893 (0.872-0.915)		1.000 (reference)		1.000 (reference)	
Conventional model +YKL-40	0.900 (0.879-0.921)	0.653	0.053 (0.005-0.101)	0.031	0.018 (0.009-0.027)	0.001
All-cause mortality						
Conventional model	0.899 (0.876-0.922)		1.000 (reference)		1.000 (reference)	
Conventional model +YKL-40	0.906 (0.884-0.927)	0.985	0.162 (0.024-0.265)	0.036	0.009 (-0.004-0.032)	0.196
Recurrent stroke						
Conventional model	0.806 (0.768-0.846)		1.000 (reference)		1.000 (reference)	
Conventional model +YKL-40	0.810 (0.771-0.848)	0.175	0.104 (-0.066-0.213)	0.144	0.005 (-0.002-0.021)	0.212

Conventional model included age, gender, history of hypertension, diabetes mellitus, hyperlipidemia, coronary artery disease, atrial fibrillation, smoking, drinking alcohol, baseline NIHSS, intravenous thrombolysis, SAP, and TOAST subtype.

Abbreviations: CI, confidence interval; IDI, integrated discrimination index; NRI, net reclassification improvement; NIHSS, National Institute of Health Stroke Scale; SAP, stroke-associated pneumonia; TOAST, Trial of Org 10172 in Acute Stroke Treatment.

## DISCUSSION

The present study showed that elevated YKL-40 levels at admission of AIS patients were statistically associated with poor outcome and all-cause mortality, but not recurrent stroke after one-year follow-up. Besides, the addition of YKL-40 to a model containing conventional risk factors could improve risk reclassification for poor outcome and all-cause mortality of AIS.

YKL-40 is getting more and more attention due to its role in mediating vascular inflammation, which might result in vascular smooth muscle cells activation and vascular endothelial cells dysfunction, and eventually contributes to the development of atherosclerosis [12]. The association between YKL-40 and ischemic stroke has been explored in previous studies. Kjaergaard et al. measured plasma YKL-40 in 21647 individuals from the Danish general population, and found that high plasma YKL-40 was associated with risks of ischemic stroke with an adjusted HR of 1.99 (95% CI 1.49-2.67) per standard deviation (SD) of YKL-40 [7]. In addition, a nested case-control study reported that high YKL-40 level was significantly associated with incident thromboembolic stroke in a population with healthy women of European ancestry [13]. These studies elucidated that YKL-40 could play a crucial role in the pathogenesis of AIS.

Limited data were available about the association between YKL-40 and long-term prognosis in AIS patients. A previous study based on 105 Korean AIS patients demonstrated that elevated YKL-40 levels showed positive associations with NIHSS and infarct volume. Meanwhile, YKL-40 in the highest tertile was also correlated with 3-month poor clinical outcome (OR 5.73, 95% CI 1.17-28.08, *P* = 0.03), compared with the lowest tertile [14]. In another study among 141 Chinese patients with large-artery atherosclerosis stroke, patients with elevated YKL-40 levels might develop poor functional outcome after 3-month follow-up (OR 6.47, 95% CI 1.36-30.76, *P* = 0.02) [15]. Of note, these studies were performed in only one single-center with a relatively small sample size, as well as relatively short-term follow-up. More importantly, recent studies on the correlation between YKL-40 and stroke recurrence have produced conflicting results. As reported by Li and colleagues, YKL-40 was an independent predictor of recurrent stroke and poor functional outcome in patients with AIS or transient ischemic attack [10]. However, another study revealed no significant association between higher YKL-40 and stroke recurrence [9]. In our study, although the fourth quartile of YKL-40 was associated with recurrent stroke in the unadjusted model, the association was not statistically significant after adjusting for potential confounders, which was consistent with findings by Qin and colleagues [9]. We speculate that the discrepancy may, in part, be attributed to different definitions of poor functional outcome and stroke recurrence, different measurements of YKL-40, as well as heterogeneous populations. Further well-designed studies with participants from different races and ethnicities are needed to draw definitive conclusion on this issue.

Serum YKL-40 has also been reported to be associated with SAP in our previous study, which was a strong factor influencing clinical outcomes of AIS patients, and patients with SAP have a higher risk of poor functional outcome [8, 11]. In our subgroup analysis, serum YKL-40 was positively associated with poor outcome and all-cause mortality in patients without SAP, while the positive association in patients with SAP did not reach statistical significance. Our study highlighted that serum YKL-40 is a reliable and valuable prognostic biomarker for clinical outcome of AIS especially those without SAP. Additionally, age, gender, stroke severity, and treatment of intravenous thrombolysis may also influence the prognosis of stroke [2, 16]. We did not identify subgroups specifically being affected by these confounding factors, indicating that serum YKL-40 is a relative independent and stable biomarker for stroke prognosis.

The precise mechanisms underlying the negative effect of elevated YKL-40 concentrations on clinical prognosis after stroke are not well clarified. Several possible explanations have been proposed. First of all, YKL-40 is a glycoprotein mainly synthesized and secreted by neutrophils as well as macrophages in inflammatory progression [5] and thus provides knowledge regarding the risk of SAP, which has been verified in our previous study [8]. Because SAP is closely associated with high mortality and morbidity after AIS [17, 18], it is not surprising that YKL-40 is also related to poor clinical prognosis. Moreover, inflammatory process is extraordinarily critical during the pathogenesis and progression of ischemic stroke, from pre-stroke arteriosclerosis to post-stroke brain damage [3, 19], among which YKL-40 is one of the most recognized components. On one hand, as an endothelial-related inflammatory mediator, increased YKL-40 may contribute to activation of endothelial cells to express vascular adhesion molecule-1 and intercellular adhesion molecule-1, further damaging vascular smooth muscle cells and endothelial cells, as well as accelerating the progression of atherosclerosis and plaque rupture [12]. On the other hand, YKL-40 expression is activated via stimulation of pro-inflammatory cytokines, such as tumor necrosis factor (TNF)-α, interleukin (IL)-1β, IL-6 and interferon-γ, which are released from ischemic penumbra after stroke and further significantly involved in the progression of neuronal necrosis and blood-brain barrier disruption [20, 21]. Additionally, in a recent study by Matute-Blanch et al., exposure of neurons to YKL-40 resulted in a significant reduction of neurite length and neuronal survival, suggesting a specific neurotoxic role of YKL-40 [22]. These may be significant explanations for poor clinical prognosis.

In recent years, YKL-40 has been proposed as a therapeutic target for the treatment of neurological diseases. An animal experiment found that the targeted deletion of YKL-40 suppressed glial phagocytic activation and promoted amyloid accumulation in a mouse APP/PS1 model of Alzheimer's disease [23]. Another study showed that the activation of adenosine A2A receptor could inhibit the expression of YKL-40 and thereby alleviate white matter injury in cerebral small vessel disease [24]. Furthermore, Bonneh-Barkay et al. have reported that absence of YKL-40 in mice resulted in more pronounced neuroinflammation and gliosis, suggesting a potential role of YKL-40 as therapeutic candidate for modulating neuroinflammation [25]. By contract, in a mouse model of stroke, YKL-40 knockout deteriorated ischemia/reperfusion damage and accelerate stroke development through enhancement of neuroinflammation [26]. Whether the modulation of serum YKL-40 concentrations within appropriate range might improve clinical outcomes after AIS is ambiguous and deserves to be further investigated.

The findings of our study should be interpreted cautiously because of several potential limitations. Firstly, quite a few AIS patients were not included due to the strict exclusion criteria and assessment, which might lead to selection bias. Secondly, serum YKL-40 levels were measured only once at admission, while dynamic measurements of YKL-40 at different time points may offer more objective and systematic prognostic information. Thirdly, it is reported that rehabilitation treatment or medication after hospitalization may reduce disability and improve activity capacity [27, 28], which were not measured in our study protocol. We cannot exclude these unmeasured confounding effects on the relationship of YKL-40 and stroke prognosis. Also, we are not able to totally exclude the underlying effect of other undetected conditions such as immunosuppression, CHI3L1 gene variants, and COVID-19 status. Finally, we did not recruit an external verification cohort to verify the association between YKL-40 and stroke prognosis. Further well-designed prospective clinical trials with long-term follow-up are required to validate our findings in the future.

## CONCLUSIONS

In summary, the present study demonstrated that serum YKL-40 revealed its prognostic value in one-year poor outcome and all-cause mortality but not stroke recurrence in Chinese AIS patients, especially in those without SAP. We cautiously asserted that serum YKL-40 might be a valuable prognostic biomarker and a potential therapeutic target for AIS.

## MATERIALS AND METHODS

### Patient selection

We conducted a prospective cohort study at the Taixing People's Hospital and Nanjing First Hospital. From February 2020 to March 2021, all patients with AIS at the stroke centers were recruited consecutively. Patients were eligible for inclusion if they were admitted within 48 hours of symptom onset. The exclusion criteria were as follows: (a) age < 18 years; (b) previous ischemic stroke or intracerebral hemorrhage; (c) pre-stroke mRS score > 1; (d) unstable conditions such as malignant tumor, heart failure, renal failure, hepatic failure, immunosuppressant treatment, or antibiotics treatment; (e) incomplete clinical data; (f) lost to follow-up during one-year.

### Clinical data

We collected clinical data including demographics (age and gender), past medical history (hypertension, dyslipidemia, diabetes mellitus, coronary artery disease, atrial fibrillation, smoking and drinking alcohol), clinical assessment (NIHSS, intravenous thrombolysis, dysphagia, SAP, stroke subtype) as well as laboratory data (leucocyte, total cholesterol [TC], TG, low-density lipoprotein [LDL], HDL, FBG, homocysteine, hs-CRP). Dysphagia was identified using a bedside non-instrumented swallowing test within the first day after admission. SAP was diagnosed according to the modified Centers for Disease Control and Prevention criteria of hospital-acquired pneumonia [29]. Stroke subtype of each AIS patient was categorized in light of TOAST criteria [30].

### Measurement of YKL-40

Blood samples were collected from all participants within the first 24 hours of admission after overnight fasting. After been centrifuged at 1,500×*g* for 15 min, serum samples were isolated and frozen at −80° C until later analysis. Serum YKL-40 levels were determined with a four-parameter curve using a commercial enzyme-linked immunosorbent assay kit (Cat No. ab255719, Abcam). Intra-panel calibration was performed in line with the manufacturer’s instructions, where the calculated minimal detectable dose is 3.9 pg/mL. The mean intra-assay and inter-assay coefficients of variation for YKL-40 were shown to be 2.4% and 1.7%, respectively.

### Clinical outcomes assessment

The participants were followed up via telephone or face-to-face at one-year after stroke onset by two trained neurologists who were blinded to baseline clinical data. Clinical outcomes included poor outcome, all-cause mortality and recurrent stroke. Poor outcome was defined as the mRS score of more than 2 (3-6). Recurrent stroke included ischemic stroke, intracerebral hemorrhage, and spontaneous subarachnoid hemorrhage.

### Statistical analysis

Continuous variables were presented as mean ± SD or medians with IQRs. Categorical variables were described as frequencies and percentages. Differences in baseline characteristics among YKL-40 quartiles were conducted using analysis of variance or the Kruskal-Wallis test for continuous variables, and Pearson’s chi-square test for categorical variables. The association between YKL-40 and poor outcome was estimated by logistic regression models, while the associations between YKL-40 and all-cause mortality as well as recurrent stroke were estimated by Cox regression models, with the first quartile of YKL-40 as a reference group. ORs or HRs with 95% CIs were displayed. Potential covariates that may be associated with clinical outcomes or YKL-40 were included in the multivariable model, which were selected on the basis of differences in baseline characteristics among YKL-40 quartiles or on the basis of theoretical considerations. These covariates included age, gender, history of hypertension, diabetes mellitus, hyperlipidemia, coronary artery disease, atrial fibrillation, smoking, drinking alcohol, baseline NIHSS, intravenous thrombolysis, dysphagia, SAP, TOAST subtype, leukocyte, TC, TG, HDL, LDL, FBG, homocysteine and hs-CRP. Additionally, C-statistic was constructed to estimate the discriminatory power of YKL-40 for clinical outcomes beyond conventional model, and NRI as well as IDI were calculated to evaluate the predictive power of YKL-40 when added to conventional model [31, 32]. Furthermore, subgroup analyses were performed to assess the potential effect of confounding factors (age, gender, baseline NIHSS, intravenous thrombolysis, and SAP) on the associations between YKL-40 and poor outcome as well as all-cause mortality. All statistical analyses were 2-tailed, and *P* value < 0.05 was considered to indicate statistical significance. Statistical analyses were conducted by R 4.1.3 (http://www.R-project.org/), SPSS version 26.0 (SPSS, Inc., Chicago, IL, USA) and GraphPad Prism version 9.3.1 (GraphPad Software, San Diego, CA, USA).
